# Core principles of responsible generative AI usage in research

**DOI:** 10.1007/s43681-025-00768-8

**Published:** 2025-10-14

**Authors:** Tim-Dorian Knöchel, Konrad J. Schweizer, Oguz A. Acar, Atakan M. Akil, Ali H. Al-Hoorie, Florian Buehler, Mahmoud M. Elsherif, Alice Giannini, Evelien Heyselaar, Mohammad Hosseini, Vinodh Ilangovan, Marton Kovacs, Zhicheng Lin, Meng Liu, Anco Peeters, Don van Ravenzwaaij, Marek A. Vranka, Yuki Yamada, Yu-Fang Yang, Balazs Aczel

**Affiliations:** 1 Department of Social and Cultural Psychology, Behavioural Science Institute, Radboud University, Nijmegen, The Netherlands; 2 Donders Institute for Brain, Cognition and Behaviour, Radboud University, Nijmegen, The Netherlands; 3 Department of Clinical Psychology, Behavioural Science Institute, Radboud University, Nijmegen, The Netherlands; 4 Department of Marketing, King’s Business School, King’s College London, London, United Kingdom; 5 Institute of Psychology, ELTE Eötvös Loránd University, Budapest, Hungary; 6 Institute of Psychology, University of Pécs, Pécs, Hungary; 7 Jubail English Language and Preparatory Year Institute, Royal Commission for Jubail and Yanbu, Jubail, Saudi Arabia; 8 Department of Business and Management, Vorarlberg University of Applied Sciences, Dornbirn, Austria; 9 Department of Psychology, University of Birmingham, Birmingham, United Kingdom; 10 School of Psychology and Vision Sciences, University of Leicester, Leicester, United Kingdom; 11 Department of Criminal Law and Criminology, Faculty of Law, Maastricht University, Maastricht, The Netherlands; 12 Department of Communication and Media, Behavioural Science Institute, Radboud University, Nijmegen, The Netherlands; 13 Department of Preventive Medicine, Northwestern University Feinberg School of Medicine, Chicago, IL, USA; 14 TIB Leibniz Information Centre for Science and Technology, Hannover, Germany; 15 Doctoral School of Psychology, ELTE Eötvös Loránd University, Budapest, Hungary; 16 MNB Institute, John von Neumann University, Kecskemét, Hungary; 17 Department of Psychology, University of Science and Technology of China, Hefei, China; 18 Department of Psychology, Yonsei University, Seoul, Republic of Korea; 19 School of English and International Studies, Beijing Foreign Studies University, Beijing, China; 20 School of Artificial Intelligence, Radboud University, Nijmegen, The Netherlands; 21 Faculty of Behavioural and Social Sciences, University of Groningen, Groningen, The Netherlands; 22 Department of Marketing Communication and PR, Institute of Communication Studies and Journalism, Charles University, Prague, Czech Republic; 23 Division of Experimental Natural Science, Faculty of Arts and Science, Kyushu University, Fukuoka, Japan; 24 Division of Experimental Psychology and Neuropsychology, Department of Education and Psychology, Freie Universität Berlin, Berlin, Germany

**Keywords:** Ethics, Generative artificial intelligence, Guidelines, Policy and regulation, Research integrity

## Abstract

In a rapidly evolving Generative Artificial Intelligence (GenAI) landscape, researchers, policymakers, and publishers have to continuously redefine responsible research practices. To ensure guidance of GenAI use in research, core principles that remain stable despite technological advancement are needed. This article defines a list of principles guiding the responsible use of GenAI in research, regardless of use case and GenAI technology employed. To define this framework, we conducted an anonymised Delphi consensus procedure comprising a panel of 16 international and multidisciplinary experts in AI, social sciences, law, ethics, and scientific publishing. After three rounds of independent rating and feedback, the panel reached consensus on eight sequentially ordered principles required for responsible GenAI usage: Regulations, Data Security, Quality Control, Originality, Bias Mitigation, Accountability, Transparency, and Broader Impact. For the clear reporting of adherence to these principles, we created a checklist allowing active implementation into the research process. With these efforts, we aim to guide everyday research, support the development of further specified regulations, policies, and guidelines, and promote discussion about GenAI use in research.

**Supplementary Information:**

The online version contains supplementary material available at 10.1007/s43681-025-00768-8.

Generative artificial intelligence (GenAI) refers to AI systems capable of producing novel content (e.g., text, images, code, or data) in response to input prompts. Powered by large language models and generative algorithms, GenAI tools have a growing impact on how research is conducted [1]. While GenAI can drastically speed up certain tasks, its use presents serious risks for research integrity, security, and may diffuse responsibility for adverse outcomes [2]. Ongoing efforts by policymakers, scientists, publishers, and research institutions aim to address these risks by formulating regulations for the responsible use of AI in research. Such efforts apply at varying scopes, from broad ethics frameworks to regional research and development guidelines, academic consensus frameworks, publication ethics standards, down to publisher and journal policies, and, finally, discipline-specific or tool-specific checklists. Some approaches refer to all forms of AI usage, while others target GenAI in particular, and further distinctions can be made on whether the regulation is descriptive or prescriptive. Although these regulatory efforts are vital in guiding research practices, the efficacy and sustainability of any regulation are significantly challenged by the continuous evolution of GenAI. The resulting sudden and unforeseen developments can disorient researchers, causing confusion about appropriate and responsible scientific practices when using GenAI. Therefore, a broad and enduring foundation for the use of GenAI in research is required, serving as a guide for regulatory efforts alongside evolving AI technologies. Accordingly, this article defines overarching principles within a framework to guide the responsible use of GenAI in research, regardless of the use case or employed model. We also provide essential preparatory steps and offer a comprehensive checklist to facilitate adherence to these principles. While this framework results from a descriptive academic consensus on GenAI, with issues, such as content originality and hallucinations being specific to it, many principles also guide the responsible use of other AI tools. To identify principles, we conducted a Delphi consensus procedure comprising a panel of 16 international and multidisciplinary experts in AI, social sciences, law, ethics, and scientific publishing. The procedure was preregistered on OSF [10.17605/OSF.IO/R4W9B]. All methodological details can be found in the supplementary text of this article [https://osf.io/uvtx5]. Consensus was reached for eight principles (Fig. 1). All principles are organised in a sequential order, starting with the most general requirements, which should be addressed first, followed by steps that are relevant only if the previous principles are satisfied. The framework distinguishes itself from higher-level ethics codes by translating broad scientific and societal values into concrete action recommendations for scientific use [3] and complements more narrow publishing and discipline-specific guidelines by defining overarching principles through an expert committee.

**Fig. 1:**
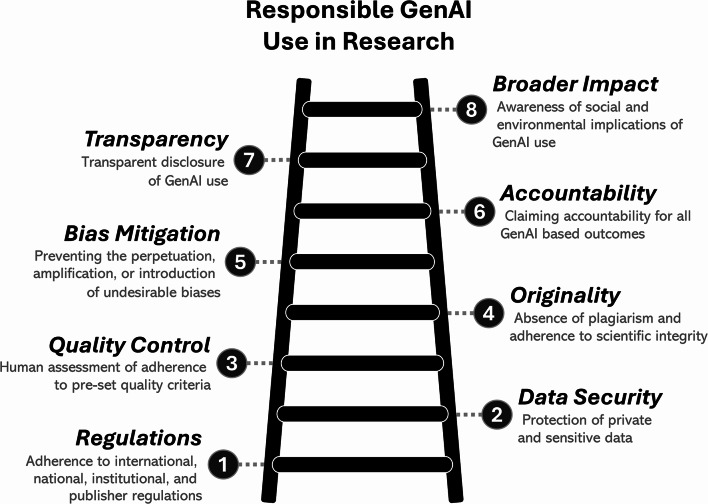
Core principles of responsible GenAI usage in research

## Regulations

Researchers must follow regulations, policies, and guidelines applicable at institutional, national, and international levels [4], as well as those set by publishers regarding their GenAI use. Ethical risk assessment and compliance with the ethical review board’s expectations help further mitigate ethical risks. In international and multi-institutional projects, differing AI regulations and ethical guidelines may apply. Ensuring that team members comply with the rules relevant to them throughout the research is critical.

## Data security

Researchers should make all reasonable efforts to ensure the security of private and sensitive data. Entering identifiable data into GenAI systems involves the risk of unauthorised third-party access, inadvertently compromising research integrity. To prevent compromising private or sensitive data, researchers can employ different strategies, such as using anonymous or pseudonymised identifiable information. Equally, identifying AI providers with more robust privacy policies and consulting data protection teams (external or institutional) is advised.

## Quality control

Like human contributions, GenAI outputs should follow quality standards to ensure good scientific practice. GenAI contributions cannot be blindly trusted, and require human verification concerning accuracy and precision (e.g., *is it correct?*); logical reasoning (e.g., *is it consistent and coherent?*); relevance (e.g., *is it topical, comprehensive, and up-to-date?*); and context-specific criteria, such as evidence standards (*professional quality*). Researchers should pre-specify procedures to verify these criteria and involve at least one human contributor to assess GenAI contributions and outputs based on the specified criteria. These criteria and verification procedures should be documented and transparently reported in any associated work.

## Originality

When using GenAI, researchers should ensure that all research components, including text in the main and supplementary documents, figures, data, and metadata, are free from plagiarism and accurately reference original sources. For instance, GenAI systems sometimes fail to provide accurate references, risking the misrepresentation of existing research. Therefore, human authors must check the originality of GenAI outputs and ensure proper acknowledgement of used sources [5]. Avoiding the direct use of GenAI outputs in publications is a cardinal way of preventing originality issues.

## Bias mitigation

Researchers should make all reasonable efforts to avoid perpetuating, amplifying, or introducing undesirable biases when using GenAI (e.g., existing gender and racial bias [6]). Uncritical reliance on GenAI can reinforce societal or academic power structures, stereotypes, or biased consensus. Researchers can employ various strategies to assess or mitigate AI biases [7]. They can consult previous evaluations of used models, or follow checklists during the implementation or dissemination of the GenAI outputs [8]. A general strategy for bias mitigation can be vetting the research using available bias benchmarks within the used AI models, domain experts, and diverse perspectives.

## Accountability

Accountability for one’s scientific work is among the hallmarks of good science and facilitates society’s trust in research results. In all published content, only humans remain accountable for the strengths and weaknesses of presented work. Unlike humans, AI systems do not make conscious decisions; they are not liable agents, and, therefore, they cannot be held accountable or sanctioned for any of their errors. Researchers who use GenAI systems should ensure that they only use models in contexts where they have sufficient expertise and information to evaluate the model’s output [9].

## Transparency

When using GenAI for research purposes, it is imperative to clearly document and communicate GenAI contributions and their validation process by humans. Acknowledging and reporting the use of GenAI tools promotes accountability, fosters trust, and facilitates verification and replication. GenAI tools are constantly being updated, but changes may not necessarily be reflected in the model version, so providing dates of usage is good practice [10]. Output may also be sensitive to the prompts [11], making their documentation informative for replication. Due to response stochasticity and iterative involvement, a complete documentation of GenAI usage may be cumbersome, and certain use cases (e.g., copy-editing) might not require detailed reporting. Field or topic-specific guidelines may be needed to ensure consistency [3].

## Broader impact

It is crucial that scientists are aware of the potential social and environmental impacts of using AI [12]. Since the training and development of AI consumes substantial energy, it produces considerable emissions [13]. When using AI, researchers should consciously consider its energy consumption. It is important to question the energy efficiency of a deployed model and to seek out more efficient options if they are able to provide comparable results. Furthermore, as GenAI will likely replace more and more areas of scientific work (e.g., data analysis, programming), researchers should pay attention to how it affects the development of their own scientific skills [14]. In addition, employing GenAI can displace or limit the involvement opportunities of co-workers, further increasing social inequalities.

**Table 1:**
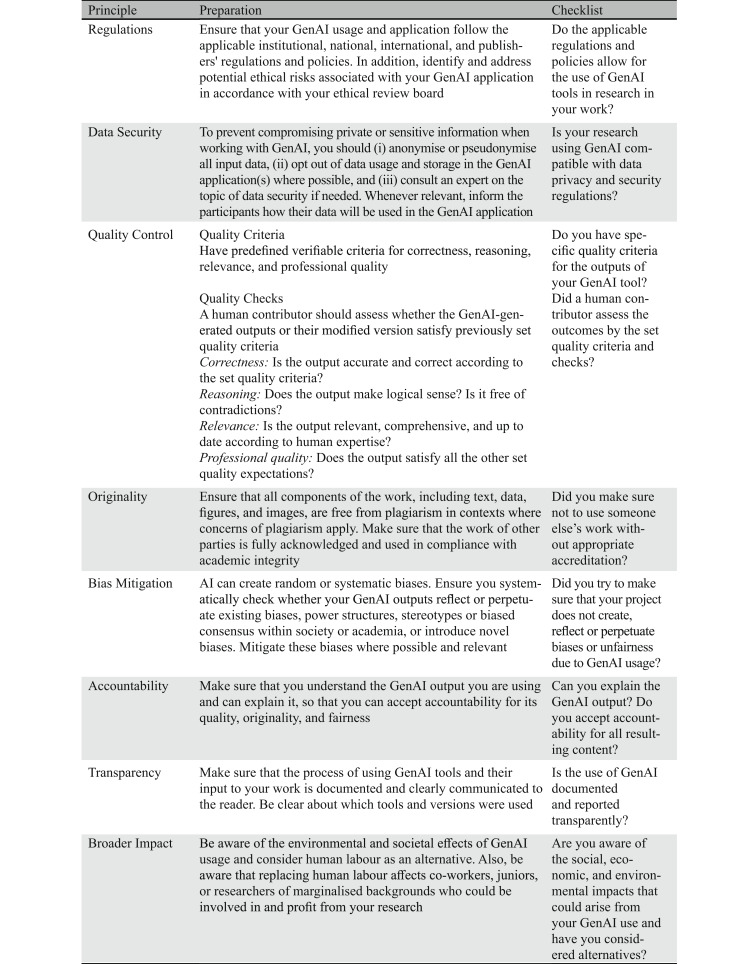
Principles, Preparation, and Checklist Questions for Responsible GenAI Usage in Research

The checklist can be filled out via a dedicated Shiny app under [https://github.com/marton-balazs-kovacs/CorePrincipleGenAIChecklist], which has been archived with Zenodo [15]. It is advised to provide a link to the generated report within a preregistration, preprint, or article.

**Checklist**

To ensure the practical applicability of these eight principles, we provide concrete preparations and checks for researchers who want to use GenAI in their work. Each item in the checklist corresponds to one of the eight principles outlined above, translating them into concrete decision points for researchers (see Table 1). It displays a framework that guides users in deciding whether a given GenAI application can be used for research purposes. This scheme includes (i) a comprehensive description of the fundamental value meant to be satisfied (principle), (ii) a short description of the steps that can be taken to satisfy the principle (preparation), and (iii) the checklist questions must be answered with ‘*yes*’ or sufficiently explained when answered with ‘*no*’, to consider the checklist to be completed. The checklist is intended to complement and support, rather than replace, a user’s own critical attitude towards responsible GenAI usage.

A collection of AI use guidance models, and their comparison is included in the supplement.

**Conclusion**

Awareness of these eight principles contributes to responsible GenAI use on both a general and concrete level. They serve as an initial take to achieve a stable guide in an ever-changing AI landscape and inform the formulation of further guidelines concerning ongoing AI developments in research.

## Electronic supplementary material

Below is the link to the electronic supplementary material.

Supplementary Material 1 (PDF 506 kb)

## Data Availability

All anonymised raw and processed data, as well as the survey materials, are publicly shared on the Open Science Framework page of this project [10.17605/OSF.IO/T3SFB]. Our methodology and data analysis plan were preregistered before the project. The preregistration document can be accessed at [10.17605/OSF.IO/R4W9B].
