# Supplementary material for: Core principles of responsible generative AI usage in research
Source: AI Ethics. 2025 Oct 14;5(6):6371–7. doi: 10.1007/s43681-025-00768-8 (PMC12592249; doi:10.1007/s43681-025-00768-8)
Supplement: Supplementary file 1 — Supplementary Material 1 (PDF 506 kb) [file 43681_2025_768_MOESM1_ESM.pdf]

Core Principles of Responsible Generative AI Usage in Research

Supplement

Tim-Dorian Knöchel[1,2*†], Konrad J. Schweizer[3*], Oguz A. Acar[4], Atakan M. Akil[5,6], Ali H. Al-Hoorie[7], Florian Buehler[8], Mahmoud Elsherif[9,10], Alice Giannini[11], Evelien Heyselaar[12], Mohammad Hosseini[13], Vinodh Ilangovan[14], Marton Kovacs[5,15,16], Zhicheng Lin[17,18], Meng Liu[19], Anco Peeters[20,2], Don van Ravenzwaaij[21], Marek A. Vranka[22], Yuki Yamada[23], Yu-Fang Yang[24], Balazs Aczel[5]

[1]Department of Social and Cultural Psychology, Behavioural Science Institute, Radboud University, Nijmegen, the Netherlands, [2]Donders Institute for Brain, Cognition and Behaviour, Radboud University, Nijmegen, the Netherlands, [3]Department of Clinical Psychology, Behavioral Science Institute, Radboud University, Nijmegen, the Netherlands, [4]Department of Marketing, King's Business School, King's College London, London, United Kingdom, [5]Institute of Psychology, ELTE Eötvös Loránd University, Budapest, Hungary, [6]Institute of Psychology, University of Pécs, Pécs, Hungary, [7]Jubail English Language and Preparatory Year Institute, Royal Commission for Jubail and Yanbu, Jubail, Saudi Arabia, [8]Department of Business and Management, Vorarlberg University of Applied Sciences, Dornbirn, Austria, [9]Department of Psychology, University of Birmingham, Birmingham, United Kingdom, [10]School of Psychology and Vision Sciences, University of Leicester, Leicester, United Kingdom, [11]Department of Criminal Law and Criminology, Faculty of Law, Maastricht University, Maastricht, the Netherlands, [12]Department of Communication and Media, Behavioural Science Institute, Radboud University, Nijmegen, the Netherlands, [13]Department of Preventive Medicine, Northwestern University Feinberg School of Medicine, Chicago, USA, [14]TIB Leibniz Information Centre for Science and Technology, Hannover, Germany, [15]Doctoral School of Psychology, ELTE Eötvös Loránd University, Budapest, Hungary, [16]MNB Institute, John von Neumann University, Kecskemét, Hungary, [17]Department of Psychology, University of Science and Technology of China, Hefei, China, [18]Department of Psychology, Yonsei University, Seoul, Republic of Korea, [19]School of English and International Studies, Beijing Foreign Studies University, Beijing, China, [20]School of Artificial Intelligence, Radboud University, Nijmegen, the Netherlands, [21]Faculty of Behavioural and Social Sciences, University of Groningen, Groningen, the Netherlands, [22]Department of Marketing Communication and PR, Institute of Communication Studies and Journalism, Charles University, Prague, Czech Republic, [23]Division of Experimental Natural Science, Faculty of Arts and Science, Kyushu University, Fukuoka, Japan, [24]Division of Experimental Psychology and Neuropsychology, Department of Education and Psychology, Freie Universität Berlin, Berlin, Germany

†Correspondence should be addressed to Tim-Dorian Knöchel; E-mail: t.d.knochel@gmail.com

*Shared first authorship

This document contains the supplementary material of the paper "Core Principles of Responsible Generative AI Usage in Research."

Preparations

Initial Guideline Items

The initial guideline items were created through an unsystematic exploration of the literature and brainstorming among eight researchers, some of whom later joined the expert panel. Subsequently, the initial guideline items were formulated based on the literature review and discussions held during several think-tank online meetings.

Delphi consensus procedure

We planned to use a version of the Delphi consensus procedure (Murphy et al., 1998), usually applied in social and medical sciences. The Delphi consensus procedure is a well-established method for obtaining expert consensus on a particular topic. It involves a group of experts participating in several rounds of structured communication to reach consensus (Sawhney et al., 2020). This approach is particularly useful in situations where limited data are available and there is an urgent need for expert guidance, as it allows for the systematic gathering of opinions from diverse groups of experts (Sawhney et al., 2020). The Delphi process typically involves multiple iterations or rounds in which participants provide feedback, allowing them to make informed decisions based on the recommendations received.

For the current consensus, we used a panel of 16 members and a total of 3 rounds. The experts were questioned and rated their questions within a Qualtrics form. We used a median equal to or larger than 6 and an interquartile range smaller or equal to 2 as

requirements for consensus on each item (Aczel et al., 2020, 2021). Note that a median rating of 6 is lower than the typical median rating of 8 in medical sciences, where Delphi procedures are frequently applied. We adopted a more lenient cutoff as methodological recommendations for the social and behavioural sciences differ somewhat from those aimed at reaching a medical consensus, where even slight hesitation warrants caution. Yet, all median agreement ratings were at 8 or higher for the final formulation of the guideline, thus also satisfying the more conservative criterion within the medical field.

Expert panel

During the project, an expert panel was surveyed. The panel consisted of 16 invited experts with relevant knowledge of using, developing, and working with artificial intelligence for research purposes. Initially, 11 experts were recruited through an announcement on X (formerly Twitter). 5 additional experts were recruited via invitation to ensure diversity across disciplines, nationalities, genders, and fields of expertise. Based on Murphy et al. (1998), we found the number of experts to be sufficient, as more than 12 experts are not necessary to further increase the reliability of consensus procedures. A description of all experts can be found in our OSF project [https://osf.io/c5w67].

Preregistration

The method and the initial survey were preregistered on April 7, 2024, under [https://doi.org/10.17605/OSF.IO/R4W9B].

Procedure

Supplement Figure 1 displays the timeline of the project.

Supplement Figure 1 | Timeline of the project

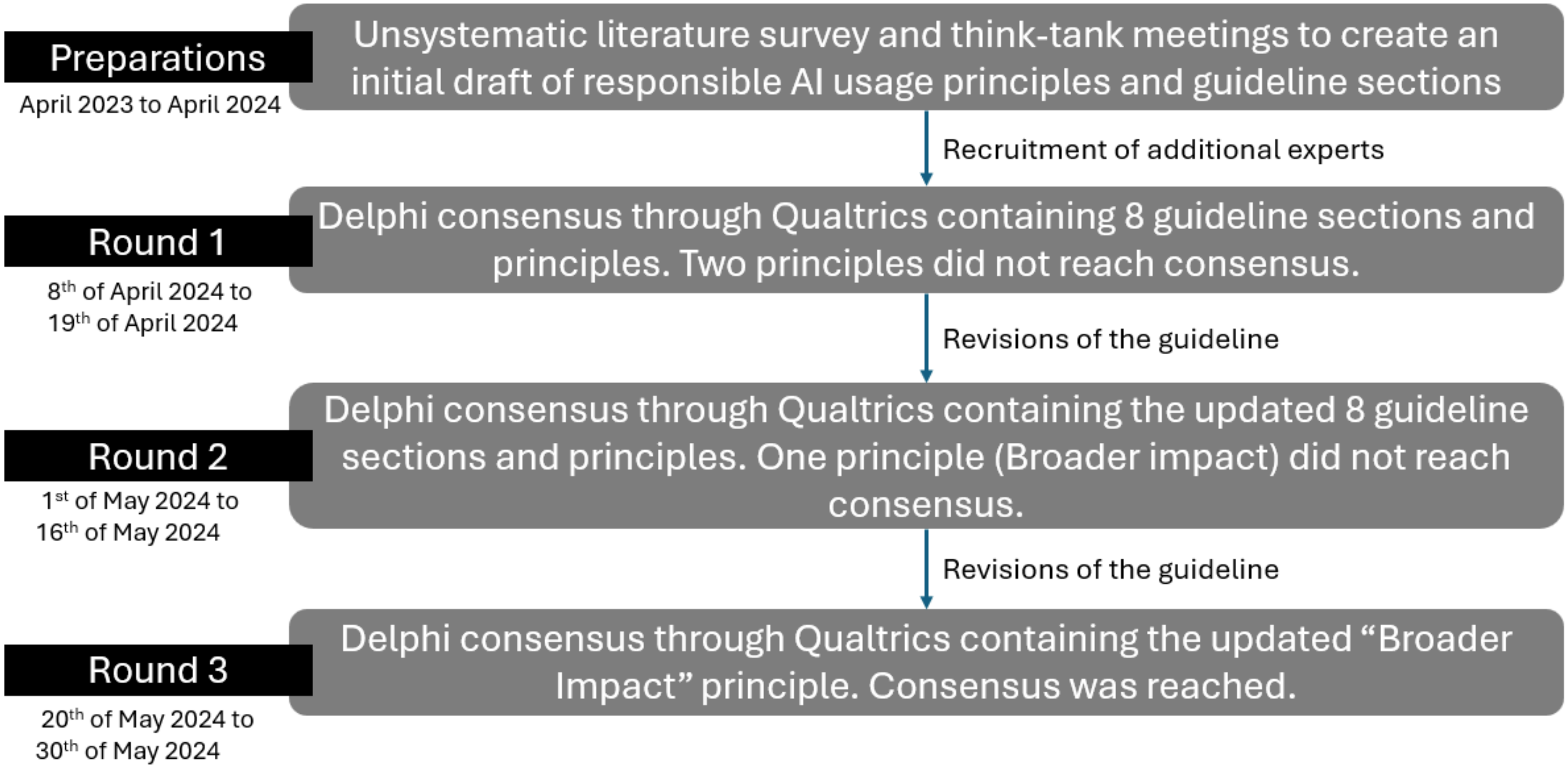

First Round

An initial guideline draft was sent to the members of the expert panel to answer the questions regarding each section of the guideline. The survey can be viewed at [https://osf.io/7d3yc]. This survey consisted of (i) an information section, (ii) consent, (iii) a 9-point Likert rating question for each guideline section ("To what degree do you agree or disagree with the content and wording of this updated guideline section?"; Likert Disagree (0) to Agree (9)), (iv) an open question for each guideline section ("In addition, regardless of your rating if you have any further suggestions, remarks or explanations, please also add them to the corresponding box"), and (v) an open question for further comments regarding the full guideline draft ("If you have any further comments or suggestions, please share them here. This can also include proposing a new structure of principles or adding another principle"). The experts were given a period of 10 days to provide their ratings.

Once the responses were collected, the project proposers used the comments and recommendations to amend and extend the guidelines. A written (also anonymous if desired) or oral discussion of the panel members was offered to resolve outstanding uncertainties about the experts' comments, but was not found to be necessary.

Following our preregistration, we regarded an item as having reached consensus in case its interquartile range was 2 or smaller, and the median rating was 6 or higher. If one section did not reach consensus or its median agreement rating was lower than 6, that section was modified by the main authors based on the received comments and sent out for a new round of the survey, repeating the above-described procedure. As written in the preregistration, small changes to the wording of guideline sections were still applied after the consensus. All changes can be seen in Supplementary Table 1. In case the modifications were beyond superficial changes of wording, items were included again for a rating, even if a consensus was reached before. Two further rounds of the surveys were sent to the members of the Expert Panel in order to reach a consensus on all guideline sections. Only those sections were eligible to enter the guideline for which consensual support was obtained with a median agreement rating of at least 6 or higher and an inter-quartile

range of the ratings of 2 or smaller (acceptance criterion). All sections reached this criterion. Only participants who responded to the first round of the survey received the materials for the subsequent round of the procedure for rating. There were no omissions.

Supplementary Table 1 | Changes of Guideline items from round one to round three

**Round 1**					**Round 2 (Changes)**					**Round 3 (Changes)**				
**Section**	**Preparation**	**Checklist item**	**Answer options**	**Consensus**	**Section**	**Preparation**	**Checklist item**	**Answer options**	**Consensus**	**Section**	**Preparation**	**Checklist item**	**Answer options**	**Consensus**
**Regulations**	Make sure that your AI usage follows the relevant national, international, institutional ethical review board, and publisher regulations and policies.	Do the relevant regulations and policies allow the use of AI tools in your work?	Yes/No<br>Open-ended	Yes	**Regulations**	Ensure that your AI usage and application follow the applicable institutional, national, international, and, if applicable, publishers' regulations and policies. In addition, identify and address potential ethical risks associated with your AI application in accordance with the ethical review board.	Do the applicable regulations and policies allow for the use of AI tools in research in your work?	Yes/No<br>Open-ended	Yes	**Regulations**	Ensure that your AI usage and application follow the applicable institutional, national, international, and publishers' regulations and policies. In addition, identify and address potential ethical risks associated with your AI application in accordance with your ethical review board.	Do the applicable regulations and policies allow for the use of AI tools in research in your work?	Yes/No<br>Open-ended	Yes

**Data Security**	To prevent compromising private or sensitive information when working with AI, you should (i) anonymize the data where possible; and (ii) involve your Data Protection Officer.	Did you secure data privacy when using AI?	Yes/No<br><br>Open-ended<br><br>Not applicable	Yes	**Data Security**	To prevent compromising private or sensitive information when working with AI, you should (i) anonymize or pseudonymize all input data, (ii) opt out of data usage and storage in the AI application(s) where possible, and (iii) involve an expert on the topic of data security. Whenever relevant, inform the participants how their data will be used in the AI application.	Is your research using AI compatible with data privacy and security regulations?	Yes/No<br><br>Open-ended<br><br>Not applicable	Yes	**Data Security**	To prevent compromising private or sensitive information when working with AI, you should (i) anonymize or pseudonymize all input data, (ii) opt out of data usage and storage in the AI application(s) where possible, and (iii) consult an expert on the topic of data security if needed. Whenever relevant, inform the participants how their data will be used in the AI application.	Is your research using AI compatible with data privacy and security regulations?	Yes/No<br><br>Open-ended<br><br>Not applicable	Yes
**Accountability**	Make sure that you understand all the AI-generated output, you will be able to explain it, and remain accountable for its quality, fairness, and originality.	Are you accountable for all the outcome created with the help of AI?	Yes/No<br><br>Open-ended	Yes	**Quality Control**<br><br>(Changed order)	**Quality Criteria**<br>Have verifiable target expectation for correctness, reasoning, relevance, and professional quality. Have the quality check process specified before the AI usage.<br><br>**Quality Checks**<br>A human contributor should assess	Do you have specific quality criteria for the outputs of your AI tool?	Yes/No<br><br>Open-ended	Yes	**Quality Control**	**Quality Criteria**<br>Have predefined verifiable criteria for correctness, reasoning, relevance, and professional quality.<br><br>**Quality Checks**<br>A human contributor should assess whether the AI-generated	Did a human contribut or assess the outcomes by the set quality criteria and checks?	Yes/No<br><br>Open-ended	Yes

						whether the AI-generated outputs or their modified version satisfy previously set quality criteria. ***Correctness:*** *Is the output accurate and correct according to the set quality criteria?* ***Reasoning:*** *Does the output make logical sense? Is it free of contradictions?* ***Relevance:*** *Is the output relevant, comprehensive, and up-to-date according to human expertise?* ***Professional quality:*** *Does the output satisfy all the other set quality expectations?*	Did a human contributor assess the outcomes by the set quality criteria and checks?				outputs or their modified version satisfy previously set quality criteria. ***Correctness:*** *Is the output accurate and correct according to the set quality criteria?* ***Reasoning:*** *Does the output make logical sense? Is it free of contradictions?* ***Relevance:*** *Is the output relevant, comprehensive, and up-to-date according to human expertise?* ***Professional quality:*** *Does the output satisfy all the other set quality expectations?*			
**Transparency**	Make sure that the process of using AI tools and their input to your work are documented and communicated according to the relevant transparency standards.	Is the use of AI documented and reported according to the relevant transparency standards?	Yes/No<br><br>Open-ended	Yes	**Originality**<br><br>(Changed order)	Ensure that all components of the work, including text, figures, and images, are free from plagiarism in contexts where concerns of plagiarism apply. Make sure that the work of other parties is fully acknowledged	Did you make sure not using someone else's work without appropriate accreditation?	Yes/No<br><br>Open-ended<br><br>Not applicable	Yes	**Originality**	Ensure that all components of the work, including text, data, figures, and images, are free from plagiarism in contexts where concerns of plagiarism apply. Make sure that the work of other parties is fully	Did you make sure not to use someone else's work without appropriate accreditation?	Yes/No<br><br>Open-ended<br><br>Not applicable	Yes

						and used in compliance with academic integrity.					acknowledged and used in compliance with academic integrity.			
**Quality Control**	**Quality Criteria**<br><br>Have verifiable target expectation for correctness, reasoning, relevance, and professional quality. Have the quality check process specified in advance.<br><br>**Quality Checks**<br><br>A human contributor should assess whether the AI-generated outputs or their modified version satisfy previously set quality criteria.<br><br>***Correctness:*** *Is the output accurate and correctness according to the set quality criteria?*<br><br>***Reasoning:*** *Does it make logical sense according to experts? Is it free of contradictions?*<br><br>***Relevance:*** *Is the output relevant, comprehensive, and up-to-date according to human expertise?*<br><br>***Professional quality:*** *Does the output satisfy all the other set quality expectations?*	Did you have specific quality criteria for the outputs of your AI tool?<br><br>Did you have specific quality criteria for the outputs of your AI tool?	Yes/No<br><br>Open-ended<br><br>Not applicable	No	**Bias Mitigation**<br><br>(Changed order)	AI can cause accidental bias. Ensure you systematically check and try to mitigate the potential reinforcement of existing power structures, stereotypes, and biased scientific consensus.	Did you assess the potential biases and unfairness that can result from AI usage in your project?	Yes/No<br><br>Open-ended<br><br>Not applicable	No	**Bias Mitigation**	AI can create random or systematic biases. Ensure you systematically check whether your AI outputs reflect or perpetuate existing biases, power structures, stereotypes or biased consensus within society or academia, or introduce novel biases. Mitigate these biases where possible and relevant.	Did you try to make sure that your project does not create, reflect or perpetuate biases or unfairness due to AI usage?	Yes/No<br><br>Open-ended<br><br>Not applicable	Yes

**Originality**	Ensure that all components of the work, including text, figures, and images, are original and free from plagiarism in contexts where concerns of plagiarism are relevant.	Did you make sure not to use someone else's work without appropriate accreditation?	Yes/No<br><br>Open-ended<br><br>Not applicable	Yes	**Accountability**<br><br>(Changed order)	Make sure that you understand the AI-generated output you are using, can explain it, and remain accountable for its quality, fairness, and originality.	Are you accountable for all the outcome created with the help of AI?	Yes/No<br><br>Open-ended	Yes	**Accountability**	Make sure that you understand the AI output you are using and can explain it, so that you can accept accountability for its quality, originality, and fairness.	Can you explain the AI output? Do you accept accountability for all resulting content?	Yes/No<br><br>Open-ended	Yes
**Bias mitigation**	AI can cause accidental bias resulting in unfair outcomes in your work. Make sure you explore this aspect of the used materials and try to mitigate it.	Did you assess the potential biases and unfairness that can result from AI usage in your project?	Yes/No<br><br>Open-ended<br><br>Not applicable	Yes	**Transparency**<br><br>(Changed order)	Make sure that the process of using AI tools and their input to your work is documented and communicated clearly in your work.	Is the use of AI documented and reported transparently?	Yes/No<br><br>Open-ended	Yes	**Transparency**	Make sure that the process of using AI tools and their input to your work is documented and clearly communicated to the reader. Be clear about which tools and versions were used.	Is the use of AI documented and reported transparently?	Yes/No<br><br>Open-ended	Yes
**Awareness**	AI systems have a huge carbon footprint. Be aware of the environmental effect of AI usage. Also, be aware that by using AI tools you save human effort but also limit the opportunity of coworkers and juniors to be involved in your research.	Are you aware of the potential effects of your AI usage on the environment?	Yes/No<br><br>Open-ended		**Sustainability**<br><br>(Changed description)	Be aware of the environmental effects of AI usage and consider human labour as an alternative. Also, be aware that replacing human labour affects coworkers, juniors, or researchers of marginalised backgrounds who could be involved in and profit from your research.	Are you aware of the social, economic, and environmental impacts that could arise from your AI use?	Yes/No<br><br>Open-ended	Yes	**Broader Impact**<br><br>(Changed description)	Be aware of the environmental and societal effects of AI usage and consider human labour as an alternative. Also, be aware that replacing human labour affects coworkers, juniors, or researchers of marginalised backgrounds who could be involved in and profit from your research.	Are you aware of the social, economic, and environmental impacts that could arise from your AI use and have you considered alternatives?	Yes/No<br><br>Open-ended	Yes

After the preparatory survey, the sections on Data Security and Broader Impact had to be revised. Supplementary Table 2 displays the ratings for the initial survey round.

Supplementary Table 2 | Ratings of the First Survey Round

Principle	Median	Inter Quartile Range	Passed
Regulations	7	2	yes
Data security	7	3.5	no
Quality Control	8.5	2	yes
Originality	8	2	yes
Bias Mitigation	8	1.5	yes
Accountability	8.5	2	yes
Transparency	7.5	2	yes
Awareness (later Broader Impact)	6	2.5	no

Note. A pass was given based on a Median rating of 6 and an interquartile range of 2 or smaller.

Round 2

Afterward, the proposed changes were implemented for the Data Security and Awareness (later Broader Impact) sections, and minor changes in wording were applied to the others. The second survey round was thereafter sent to all Experts and can be seen at [https://osf.io/edpc3]. All sections reached consensus except for Bias mitigation, with a median rating of 8 but a high interquartile range of 2.75, scoring above the pre-defined consensus criterion. Furthermore, we added an instruction section to the survey, which was utilised in the shiny app (https://github.com/marton-balazs-kovacs/CorePrincipleGenAIChecklist).

Round 3

A third round, only containing the updated Bias Mitigation section, was sent out. The form can be seen at [https://osf.io/d9xtu]. In this final round, the section for Bias Mitigation passed the acceptance criterion with a median rating of 9 and an interquartile range of 1. Supplementary Table 3 displays the final ratings for all guideline sections.

Supplementary Table 3 | Final Ratings for All Guideline Sections

Principle	Median	Interquartile Range	Passed
Regulations	9	1	yes
Data security	8.5	1	yes
Quality Control	8	2	yes
Originality	8.5	1.75	yes
Bias Mitigation	9	1	yes
Accountability	8.5	1	yes
Transparency	9	0	yes
Broader Impact	8	1.75	yes

Note. The rating of the Bias Mitigation section was given in the third round of the survey.

Deviations from preregistration

There were no deviations from the preregistration.

Comparison to Other Current AI Use Guidance Models

The responsible use of AI in research is currently addressed by a number of frameworks and policies that operate at different levels, from international ethics guidelines (e.g., OECD, 2025; UNESCO, 2025) to journal (e.g., Elsevier, 2025; Science, 2025; Springer, 2025) and field-specific guidelines (e.g., Liu et al., 2020; Tejani et al., 2024). Despite their differing scopes, these initiatives exhibit common themes, evident through the similar overarching principles or action recommendations they use.

Nearly all frameworks and policies addressing the responsible use of AI, stress human accountability for AI outputs, following the tenet of AI as a tool, not an author or independent agent. This tenet can be seen in different forms, ranging from non-binding suggestions to take accountability for AI outputs (UNESCO, 2025), to enforced principles during publishing by prohibiting AI-generated text and authorship of AI (e.g., COPE, 2023; Nature, 2025). Transparency is also universally emphasised, underlining the disclosure of when and how AI is used, varying between general (e.g., European Parliament, 2023), tool (e.g., Cacciamani et al., 2023), and field-specific (e.g., International Committee of Medical Journal Editors, 2023) recommendations. Additionally, research integrity as a whole, including the originality of ideas, outputs and the prevention of plagiarism and fabrication as well as the formulation of quality control standards are frequently highlighted in frameworks focusing on AI (e.g., Elsevier, 2025; International Association of Scientific, Technical & Medical Publishers, 2025; Science, 2025; Springer, 2025). At the same time, bias and fairness are commonly included in broad-scale AI frameworks (e.g., European Parliament, 2023; OECD, 2025; UNESCO, 2025). However, there is also some divergence between models. While environmental impact is mentioned within larger ethics frameworks, it is not commonly addressed by publisher policies. Moreover, stakeholder engagement and public communication (Hosseini et al., 2024) stressing the need to communicate with and involve populations and stakeholders impacted by AI decision-making, is not frequently mentioned. Furthermore, calls for AI detection tools and their integration are mentioned on a field-specific policy level (e.g., World Association of Medical Editors, 2023). Yet, they are not often integrated into higher-level frameworks.

Moreover, the scope of existing AI use guidance models varies from broad to narrow and from AI in general to generative or even tool-specific recommendations. Guidance ranges from broad AI ethics spanning domains such as education, health, and environment (UNESCO, 2025), cross-sector general purpose and generative AI (OECD, 2025), AI overall in scientific research (e.g., Hosseini et al., 2024), publishing with generative AI (e.g., Liu et al., 2020; World Association of Medical Editors, 2023) to specific reporting of generative AI tools, such as ChatGPT (Cacciamani et al., 2023). Most of these frameworks are, however, not binding and take more of an advisory role. Finally, current AI use guidance models target different populations. Target populations range from international, governmental, and cross-disciplinary research targets, to industry, publisher, and editorial levels and the individual researcher level.

As the outcome of a Delphi consensus procedure, our current framework distinguishes itself from high-level ethics codes such as UNESCO´s recommendation on the ethics of AI or the OECD AI principles by translating broader societal values into a practically applied checklist for AI usage in a research context. At a regional level, our framework shares the commitment to accountability, security, and transparency with the EU AI Act, yet is more targeted towards the topic of research. Furthermore, our framework complements publication ethics and journal-specific AI use guidance, by being a more flexible guide to AI usage decisions than sheer prohibition.

Our AI use framework is a non-binding guide to responsible GenAI usage, specifically targeting researchers across disciplines. Yet, legislative guidance remains part of our framework, as our first principle requires adherence to institutional, national, and international regulations. Finally, while our framework spans the responsible use of all forms of AI used in research, it is particularly pressing in GenAI contexts, as highlighted in the manuscript.

Supplementary References

Aczel, B., Szaszi, B., Nilsonne, G., van den Akker, O. R., Albers, C. J., van Assen, M. A., Bastiaansen, J. A., Benjamin, D., Boehm, U., Botvinik-Nezer, R., Bringmann, L. F., Busch, N. A., Caruyer, E., Cataldo, A. M., Cowan, N., Delios, A., van Dongen, N. N., Donkin, C., van Doorn, J. B., … Wagenmakers, E.-J. (2021). Consensus-based guidance for conducting and reporting multi-analyst studies. *eLife*, *10*, e72185. https://doi.org/10.7554/eLife.72185

Aczel, B., Szaszi, B., Sarafoglou, A., Kekecs, Z., Kucharský, Š., Benjamin, D., Chambers, C. D., Fisher, A., Gelman, A., Gernsbacher, M. A., Ioannidis, J. P., Johnson, E., Jonas, K., Kousta, S., Lilienfeld, S. O., Lindsay, D. S., Morey, C. C., Munafò, M., Newell, B. R., … Wagenmakers, E.-J. (2020). A consensus-based transparency checklist. *Nature Human Behaviour*, *4*(1), 4–6. https://doi.org/10.1038/s41562-019-0772-6

Cacciamani, G. E., Collins, G. S., & Gill, I. S. (2023). ChatGPT: Standard reporting guidelines for responsible use. *Nature*, *618*(7964), 238–238. https://doi.org/10.1038/d41586-023-01853-w

COPE. (2023, February 13). *Authorship and AI tools*. COPE: Committee on Publication Ethics. https://publicationethics.org/guidance/cope-position/authorship-and-ai-tools

Elsevier. (2025, May 21). *Generative AI policies for journals*. Generative AI Policies for Journals. https://www.elsevier.com/about/policies-and-standards/generative-ai-policies-for-journals

European Parliament. (2023, August 6). *EU AI Act: First regulation on artificial intelligence*. Topics | European Parliament. https://www.europarl.europa.eu/topics/en/article/20230601STO93804/eu-ai-act-first-regulation-on-artificial-intelligence

Hosseini, M., Resnik, D. B., & Holmes, K. (2023). The ethics of disclosing the use of artificial intelligence tools in writing scholarly manuscripts. *Research Ethics*, *19*(4), 449–465. https://doi.org/10.1177/17470161231180449

Hosseini, M., Resnik, D. B., & Holmes, Kristi. (2024). The ethics of using artificial intelligence in scientific research: New guidance needed for a new tool. *AI and Ethics*. https://doi.org/10.1007/s43681-024-00493-8

International Association of Scientific, Technical & Medical Publishers. (2025, April 21). New STM Draft Report: Classifying AI Use in Manuscript Preparation. *STM Association*. https://stm-assoc.org/new-stm-draft-report-classifying-ai-use-in-manuscript-preparation/

International Committee of Medical Journal Editors. (2023, May). *ICMJE | News & Editorials*. Up-Dated ICMJE Recommendations. https://www.icmje.org/news-and-editorials/updated_recommendations_may2023.html

Liu, X., Cruz Rivera, S., Moher, D., Calvert, M. J., & Denniston, A. K. (2020). Reporting guidelines for clinical trial reports for interventions involving artificial intelligence: The CONSORT-AI extension. *Nature Medicine*, *26*(9), 1364–1374. https://doi.org/10.1038/s41591-020-1034-x

Murphy, M. K., Black, N. A., Lamping, D. L., McKee, C. M., Sanderson, C. F., Askham, J., & Marteau, T. (1998). Consensus development methods, and their use in clinical guideline development. *Health Technology Assessment (Winchester, England)*, *2*(3), i–iv, 1–88.

Nature. (2025, May 21). *Artificial Intelligence (AI) | Nature Portfolio*. Artificial Intelligence (AI). https://www.nature.com/nature-portfolio/editorial-policies/ai

OECD. (2025, May 21). *OECD Legal Instruments*. Recommendation of the Council on Artificial Intelligence. https://legalinstruments.oecd.org/en/instruments/oecd-legal-0449

Sawhney, M. S., Bilal, M., Pohl, H., Kushnir, V. M., Khashab, M. A., Schulman, A. R., Berzin, T. M., Chahal, P., Muthusamy, V. R., Varadarajulu, S., Banerjee, S., Ginsberg, G. G., Raju, G. S., & Feuerstein, J. D. (2020). Triaging advanced GI endoscopy procedures during the COVID-19 pandemic: Consensus recommendations using the Delphi method. *Gastrointestinal Endoscopy*, *92*(3), 535–542. https://doi.org/10.1016/j.gie.2020.05.014

Science. (2025, May 25). *Change to policy on the use of generative AI and large language models*. Editorial Policies. https://www.science.org/content/blog-post/change-policy-use-generative-ai-and-large-language-models

Springer. (2025, May 21). *Artificial Intelligence (AI) | Springer—International Publisher*. Artificial Intelligence (AI). https://www.springer.com/gp/editorial-policies/artificial-intelligence--ai-/25428500

Tejani, A. S., Klontzas, M. E., Gatti, A. A., Mongan, J. T., Moy, L., Park, S. H., & Kahn, C. E. (2024). Checklist for Artificial Intelligence in Medical Imaging (CLAIM): 2024 Update. *Radiology: Artificial Intelligence*, *6*(4), e240300. https://doi.org/10.1148/ryai.240300

UNESCO. (2025, May 21). *Recommendation on the Ethics of Artificial Intelligence—UNESCO Digital Library*. Ecommendation on the Ethics of Artificial Intelligence. https://unesdoc.unesco.org/ark:/48223/pf0000381137

World Association of Medical Editors. (2023, October 25). *WAME Revised Recommendations on Chatbots and Generative AI || WAME*. https://www.wame.org/news-details.php?nid=40
